# Group B *Streptococcus* and diabetes: Finding the sweet spot

**DOI:** 10.1371/journal.ppat.1011133

**Published:** 2023-02-16

**Authors:** Rebecca A. Keogh, Kelly S. Doran

**Affiliations:** Department of Immunology and Microbiology, University of Colorado Anschutz, Aurora, Colorado, United States of America; University of Geneva: Universite de Geneve, SWITZERLAND

## Introduction

*Streptococcus agalactiae* (Group B *Streptococcus*, GBS) is a gram-positive pathobiont that colonizes the gastrointestinal and urogenital tracts of approximately 25% of individuals. GBS also commonly colonizes the female reproductive tract and during pregnancy can be transmitted to the fetus or newborn, where it is a leading cause of neonatal infection including pneumonia, sepsis, and meningitis. However, numerous reports have documented the increased frequency of GBS invasive infection in nonpregnant adults who have underlying conditions such as diabetes mellitus, cancer, and HIV [1–5]. A survey in the United States from 1990 to 2007 found that 88% of individuals with GBS infections had at least 1 underlying medical condition with diabetes being the highest [2]. Another study over 2008 to 2016 found an increase in invasive GBS infections in nonpregnant adults, with obesity and diabetes being the highest risk factors [1]. While there is a spectrum of GBS infections in adults, primary bacteremia and skin and soft-tissue infections are the most frequently reported [5]. Recent studies have correlated the use of prophylactic antibiotics to an expansion of certain virulent GBS clonal complexes (mainly CC-17) leading to more late onset disease in newborns [6]. The bacteria isolated from diabetic wound infections are difficult to treat in part due to high levels of antibiotic resistance [7]; however to date, we are unaware of a study that has examined the contribution of antibiotic usage to invasive infections in diabetic adults. These findings highlight the importance of studying GBS infection during diabetes. Multiple studies have used a murine model of streptozotocin-induced diabetes to demonstrate that diabetic mice are more susceptible to lethal GBS infection [8], as well as GBS sepsis, arthritis, and urinary tract infection [9,10]. Recent studies using *lepr*^*db*^ mice to model diabetic infection showed that diabetic mice are more susceptible to GBS skin wound infection and that GBS triggers a hyper-inflammatory environment in the wound [11]. Here, we review current understanding of how GBS may thrive in the diabetic environment, highlighting the impact of glucose, GBS gene regulation, and immune disfunction in diabetic individuals.

## Impact of hyperglycemia on GBS metabolism

Diabetes mellitus is a group of metabolic disorders that affects an estimated 500 million people worldwide [12]. A hallmark of diabetes is elevated blood sugar due to the inability of the body to properly utilize glucose. GBS is a facultative anaerobe and lactic acid-producing bacterium that utilizes glucose as its primary carbon source [13]. Fermentation was assumed to be the only means of GBS metabolism, as GBS lacks essential enzymes to undergo respiration [14]. However, metabolic respiration in GBS has been observed with supplementation of both heme and quinones, demonstrating that GBS may undergo respiration if it acquires these components from the environment [14,15]. Interestingly, GBS genes involved in heme homeostasis as well as electron transport and respiration were up-regulated during wound infection of diabetic mice compared to non-diabetic mice [11]. GBS may be able to acquire these enzymes from the diabetic environment or other pathogens as was shown recently between *Staphylococcus aureus* and *Enterococcus faecalis* that share metabolites during biofilm growth [16]. The ability of GBS to shift its metabolism has been hypothesized to assist the bacterium in surviving such diverse niches [14], which could be the case during diabetic infection. In addition, respiration metabolism leads to the accumulation of the major GBS nuclease NucA, which contributes to degradation of neutrophil extracellular traps (NETs) and virulence in the lung [17] and is speculated to promote GBS survival in the diabetic wound [11], although this has not yet been shown.

Glucose supplementation also enhances GBS adhesion and proliferation during biofilm formation [18]. However, D’Urzo and colleagues found that this increase in biomass is more likely due to an acidification of the medium driven by glucose-mediated fermentation [19]. While the authors did replicate the increase of GBS biofilm formation with supplementation of glucose, the acidification of medium was enough to augment biofilm formation, even in the absence of glucose [19]. The consequence of possible glucose-dependent biofilm formation is particularly intriguing and whether it contributes to GBS diabetic infection requires further study. Collectively, excess glucose could promote GBS metabolic adaptation that allows for better niche establishment and survival in the diabetic host.

## GBS regulation in diabetic infection

To determine how GBS responds to elevated glucose, Di Palo and colleagues performed transcriptomic analysis on GBS grown in medium without or with glucose (55 mM) for 30 min [20]. Approximately 27% of all genes had altered transcription in the resulting comparison, with multiple dysregulated genes being under control of the well-studied GBS two-component system CovRS, which represses numerous virulence factors [21]. Additionally when GBS was grown in human urine with or without supplementation of glucose (300 ml/dl), it was observed that glucose supplementation led to increased expression of *covRS*, as well as increased expression of virulence-related genes including the *cylE* gene that is responsible for GBS hemolytic capacity and pigment [22]. Interestingly, *cylE* assists in GBS survival against oxidative damage, which may be important in the diabetic wound environment that has high extracellular reactive oxygen species (ROS) [23,24].

Dual RNA-sequencing analysis of GBS in murine diabetic wound infection demonstrated the up-regulation of virulence factors that are repressed by CovR, including the surface plasminogen-binding protein PbsP, the nuclease NucA, and the *cyl* operon [11]. Deletion of *pbsP* and *cylE* resulted in attenuation of the bacterium in diabetic wound infection; however, these genes were dispensable in non-diabetic infection [11]. It was also found that GBS acquires spontaneous mutations in *covR* during murine diabetic wound infection, which did not occur in non-diabetic wounds [11]. The diabetic environment may therefore be enhancing GBS virulence factor production, allowing it to persist in diabetic individuals.

These transcriptomic studies demonstrate a possible link between excess glucose and altered *covRS* regulation. One possible explanation is that the signal for CovS, the sensor histidine kinase, is less abundant or different in the presence of glucose, or that CovS signaling is somehow blocked. Little is known in GBS of the extracellular signal(s) that CovS senses; however, in *Streptococcus pyogenes* (Group A *Streptococcus*, GAS), the antimicrobial peptide LL-37 directly binds CovS leading to increased expression of bacterial virulence factors [25]. RNA-seq of murine-infected wounds reveals that GBS infection results in the up-regulation of antimicrobial peptides [11], and Patras and colleagues found increased cathelicidin in the bladders of diabetic mice infected with GBS in comparison to non-diabetic [10]. Whether the presence of these antimicrobial peptides is altering CovRS regulation in GBS has yet to be determined.

Santi and colleagues found that CovRS gene regulation in GBS is altered in response to environmental pH, with many CovRS regulated genes down-regulated under acidic pH and induced in a more neutral pH [26]. They hypothesize that GBS CovRS responds to changes in pH from the acidic environment of the vagina to the more neutral pH of the infant lung resulting in increased virulence factor production [26]. Interestingly, the skin is acidic, and this acidity is key to skin barrier function [27]. When the skin is wounded, deeper tissue is exposed, leading to a more neutral pH [27]. Further, the skin of diabetic individuals has been found to have a higher pH than that of healthy controls [28]. Therefore, the more alkaline pH in infected diabetic wounds could affect CovRS regulation leading to increased virulence factor production during GBS infection.

### Impaired neutrophil response in diabetic individuals

The immune system of diabetic individuals has been well studied, as type 1 diabetes is considered an autoimmune disease and high inflammation is thought to contribute to the development of type 2 diabetes [29]. In addition, lymphocytes and myeloid cells, particularly neutrophils, have all been implicated in type 2 diabetes pathogenesis [30]. Neutrophils are key players in innate immunity that help to combat bacterial infections, and neutrophil function is greatly altered in diabetic individuals, reviewed extensively by Dowey and colleagues [12]. Glucose is the primary source of energy for neutrophils, and persistent hyperglycemia causes a shift from glycolysis to the polyol and hexosamine pathway to prevent toxic intracellular levels of glucose [31]. This shift in metabolism causes enhanced cytokine generation and production of proinflammatory genes that can be detrimental to the host [32]. Evidence for neutrophil dysfunction in GBS clearance was documented by Baker and colleagues, who found that serum from diabetic individuals had reduced opsonophagocytosis, and Mazade and colleagues who found that neutrophils grown in hyperglycemic conditions exhibited a decreased ability to phagocytose GBS [33,34].

Other hallmarks of neutrophil dysfunction during diabetes are increased NETosis, extracellular ROS production, and protease production [12,24,35,36]. Each of these processes further promotes inflammation and inhibit the successful re-establishment of the extracellular matrix therefore inhibiting wound healing and closure [37]. The fact that GBS infections are associated with higher inflammation in multiple models of diabetes [9–11] suggests that GBS has adapted to take advantage of this immune dysfunction for enhanced survival, possibly through CovRS regulation and enhanced virulence as documented above. However, additional work is necessary to determine the mechanisms by which GBS survives and exacerbates disease in highly inflammatory diabetic niches.

## Conclusions

**Fig 1:**
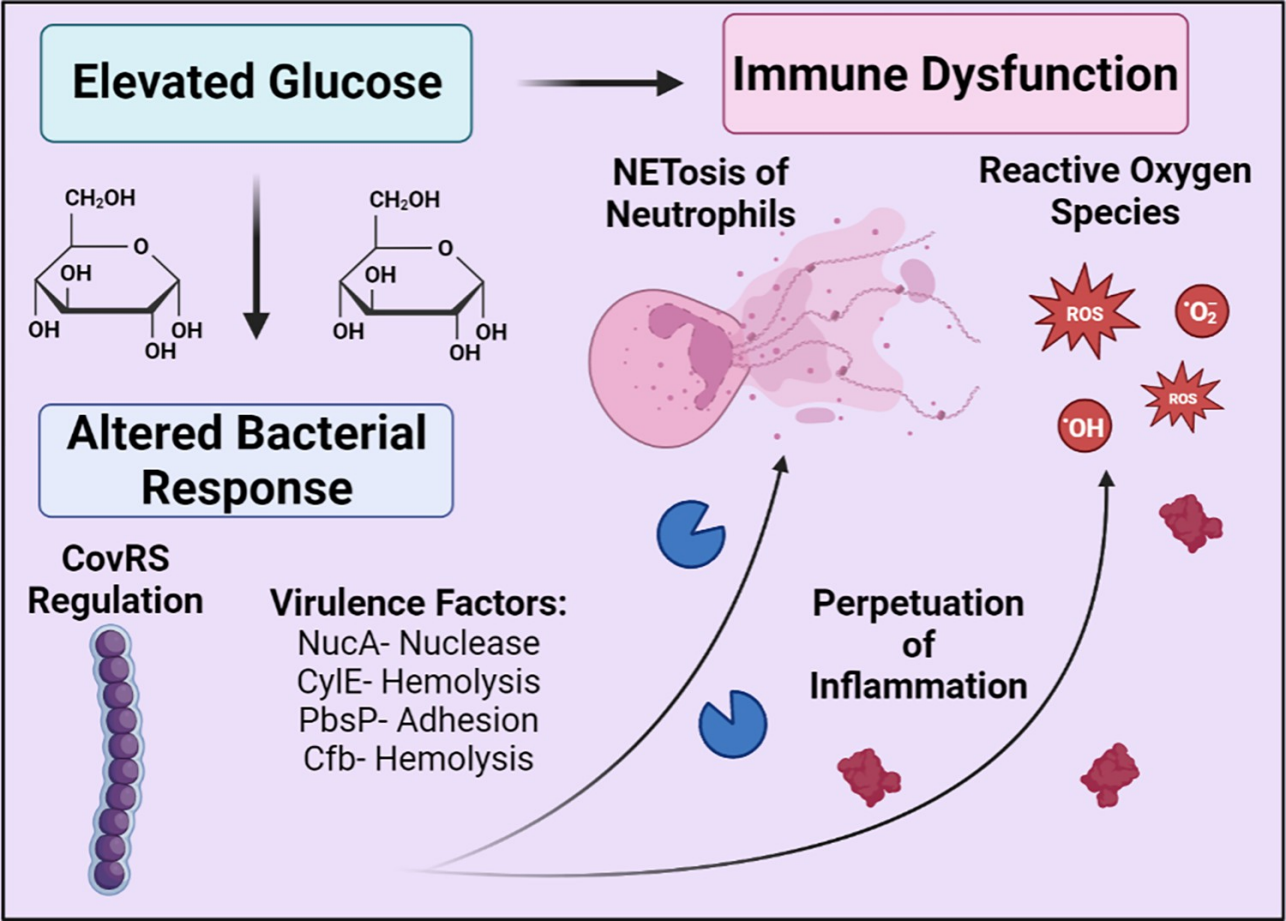
Summary of GBS in diabetic infection.

Epidemiological studies demonstrate an increased incidence of GBS in nonpregnant adults, with diabetes being the most common comorbidity. However, little is known about how GBS persists and exacerbates diabetic infection. It is likely that the diabetic environment, particularly with excess glucose and inflammation, shifts GBS metabolism and alters gene expression (**Fig 1**). This regulation appears to be largely under the control of the two-component system CovRS, which mediates the switch between a commensal and a virulent lifestyle. GBS alters its transcriptome in both exposure to glucose as well as in murine diabetic wounds, increasing expression of virulence factors [11,20]. Additionally, GBS seems to promote and survive hyperinflammation and increased immune infiltrate in multiple murine models of infection (**Fig 1**). Further investigation of GBS pathogenesis in diabetic individuals is critical to combat this bacterium in immunocompromised individuals.
